# Fzd7/Wnt7b signaling contributes to stemness and chemoresistance in pancreatic cancer

**DOI:** 10.1002/cam4.3819

**Published:** 2021-05-02

**Authors:** Zhongbo Zhang, Yuanhong Xu, Chenghai Zhao

**Affiliations:** ^1^ Department of Pancreatic and Biliary Surgery The First Hospital of China Medical University Shenyang P.R. China; ^2^ Department of Pathophysiology Basic Medical College China Medical University Shenyang P.R. China

**Keywords:** chemoresistance, Fzd7/Wnt7b, pancreatic cancer, stemness, Wnt signaling pathway

## Abstract

Mining databases and data obtained from assays on human specimens had shown that Fzd7 is closely associated with Wnt7b, that Fzd7/Wnt7b expression is upregulated in pancreatic cancer tissues compared with normal tissues, and its expression is negatively correlated with survival. Fzd7/Wnt7b knockdown in Capan‐2 and Panc‐1 cells reduced the proliferative capacity of pancreatic cancer stem cells (PCSCs), reduced drug resistance, decreased the percentage of CD24^+^CD44^+^ subset of cells and the levels of ABCG2, inhibited cell‐sphere formation, and reduced gemcitabine (GEM) resistance. In contrast, Fzd7/Wnt7b overexpression increased the percentage of the CD24^+^CD44^+^ subset of cells, and increased the levels of ABCG2 detected in cell spheroids. The gem‐resistant cells exhibited higher levels of Fzd7/Wnt7b expression, an increased percentage of CD24^+^CD44^+^ cells, and higher levels of ABCG2 compared with the parental cells. Taken together, Fzd7/Wnt7b knockdown can reduce PDAC cell stemness and chemoresistance by reducing the percentage of CSCs. Mechanistically, Fzd7 binds with Wnt7b and modulates the levels of β‐catenin, and they may exert their role via modulation of the canonical Wnt pathway.

## Graphical abstract

This study aims to elucidate the regulatory mechanism of stemness and chemoresistance in pancreatic cancer, especially the role of Wnt signaling pathway on stemness and chemoresistance of pancreatic cancer. Fzd7/Wnt7b can regulate the proportion of CSC subset, so that they influence stemness and chemoresistance.

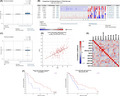

## 1 INTRODUCTION

Pancreatic cancer has a notably high mortality rate; the overall survival rate for pancreatic cancer is only 8%.^1^ Pancreatic ductal adenocarcinoma (PDAC) accounts for 90% of all pancreatic malignancies.^2^ Early pancreatic cancer is usually asymptomatic, and there is a lack of biomarkers and sensitive diagnostics for early detection, thus, the majority of pancreatic cancer patients cannot undergo surgery, as the malignancy is often detected at a more advanced stage.^3^ Although gemcitabine (GEM) has been used as the first‐line chemotherapeutic for management of advanced PDAC for several years, it is only effective in 23.8% of PDAC patients, and patients may develop gem‐resistant cells.^4^, ^5^

It has been extensively demonstrated that tumors are composed of different cell subsets, of which a small subset exhibits stem cell‐like properties, including maintaining an undifferentiated phenotype, with self‐renewal capacity, varied differentiation potential, and increased migratory capacity.^6^, ^7^ This cell subset is termed cancer stem cells (CSCs).^6^, ^7^ Pancreatic CSCs (PCSCs) were first reported in 2007.^8^ Resistance to chemotherapy and radiotherapy was consistently observed in both solid tumors and leukemic CSCs.^9^, ^10^ PCSCs exhibit strong chemoresistance and can survive conventional chemotherapy.^11^, ^12^, ^13^ This resistance underlies the high rate of recurrence and metastasis in a large percentage of patients with PDAC who have undergone operative chemotherapy. Therefore, elimination of CSCs to reduce resistance has been suggested as a novel therapeutic strategy for management of pancreatic cancer.^10^, ^11^, ^12^, ^13^

It has been demonstrated that there are a series of specific surface molecules on CSCs, for example, CD24,^14^, ^15^ CD44,^14^, ^15^, ^16^ and ABCG2,^17^, ^18^, ^19^ among others. As a drug transporter, ABCG2, has a strong ability to efflux various chemotherapeutic drugs, and is considered the effector of chemoresistance across multiple types of tumors.^17^, ^18^, ^19^ CSCs express high levels of ABC transporters,^20^ thus, CSCs can survive following chemotherapy and promote cancer relapse and resistance to treatment.

CD44,^21^ CD24,^22^ and the ABC cassette genes,^23^, ^24^ as well as several other surface markers are direct targets of Wnt pathway. The Wnt/β‐catenin signaling pathway is crucial for maintenance of CSCs; abnormal Wnt signaling can promote resistance to apoptosis and maintenance of CSCs.^25^, ^26^, ^27^, ^28^

In view of the important role of the Wnt signaling pathway on the biological behavior of CSCs, it was hypothesized that the Wnt signaling pathway may serve as an important regulator of stemness. In order to verify our hypothesis, we performed the present study to elucidate the upstream molecular pathways involved in regulation of PCSCs in pancreatic cancer, potentially providing a novel approach for future treatment of pancreatic cancer and novel targets for pharmacological research.

## 2 MATERIALS AND METHODS

### 2.1 Bioinformatics

The Oncomine Cancer Microarray database (http://www.oncomine.org) was used to study gene expression of Fzd7/Wnt7b in pancreatic cancer samples, and comparison analysis on expression of various Frizzled receptors in pancreatic adenocarcinoma was performed in Oncomine also. Gene expression data were obtained from the datasets “TCGA Pancreas”. The correlation analysis on expression of FZD7 and various Wnt proteins was performed using data from Cancer Cell Line Encyclopedia (CCLE) database (https://portals.broadinstitute.org/ccle). The univariate analysis of survival within the pancreatic cancer dataset of “TCGA‐178” and “Zhang‐90” was performed using the Kaplan–Meier analysis module of the R2 microarray analysis and visualization platform (http://r2.amc.nl). Correlation analysis of Fzd7 and Wnt7b was performed on the “tcga‐195” dataset. Correlation analysis of CTNNB1/TCF4 and Fzd7/Wnt7b was performed on the “TCGA‐178” dataset.

### 2.2 Pancreatic cancer specimen

The human tissues used in the present study were obtained from the Department of Pancreatic and Biliary Surgery in the First Hospital of China Medical University, and their use was approved by the Hospital Ethics Committee for Scientific Research. A total of 37 samples of pancreatic cancer tissues and their corresponding normal paracancerous pancreatic tissues were collected between the end of 2015 and the beginning of 2020. According to the AJCC pancreatic cancer staging standard 8th Edition, all patients were in TNM stage Ⅰa‐Ⅱb, and had undergone radical resection. Each patient was diagnosed with PDAC by two senior pathologists. During the follow‐up, apart from those lost to follow‐up (whose outcomes remain unknown), all patients succumbed to the advanced pancreatic cancer, without any other cause of death or accidental death. The clinicopathological information of patients and the relationship with pathological results are shown in Table 1.

**TABLE 1:** The relationship between Fzd7/Wnt7b and clinical pathological features in PDAC patients

	Samples	Fzd7 expression *n* (%)		Wnt7b expression *n* (%)			
	(n)	Negative	Positive	*p* value^a^	Negative	Positive	*p* value^a^
Sex				0.4624			1.0
Male	20	6	14		10	10	
Female	17	3	14		9	8	
Age^b^				1.0			0.1031
≦55	20	5	15		13	7	
>55	17	4	13		6	11	
Histological grade				0.028^c^			0.4829
Well	8	5	3		3	5	
Moderate	13	2	11		6	7	
Poor	16	2	14		10	6	
TNM stage (AJCC)				0.8769			0.4163
ⅠA	10	3	7		3	7	
ⅠB	10	2	8		5	5	
ⅡA	7	1	6		5	2	
ⅡB	10	3	7		6	4	

^a^ X^2^ test.

^b^ Media age.

^c^ Significant, *p* < 0.05.

### 2.3 Immunohistochemistry (IHC) and staining

IHC staining was performed using the standard streptavidin‐biotin‐peroxidase complex method. Briefly, 4–6 μm paraffin sections were dewaxed and rehydrated using xylol and a descending series of alcohol solutions. Endogenous peroxidase activity was blocked using 3% hydrogen peroxide for 20 min. Sections were heated for antigen retrieval in a microwave oven for 10 min in 10 mM citrate buffer, pH 6.0. Sections were subsequently incubated with primary antibodies overnight at 4℃ (anti‐Fzd7, 1:500, Abcam, cat. no. ab64636; or anti‐Wnt7b, 1:100, R&D Systems, cat. no. AF3460) in a humidified box. The following day, the samples were incubated with the appropriate biotinylated secondary antibodies for 30 min at room temperature. After washing, the results were visualized using Diaminobenzidine (Boster Biological Technology), and the sections were counter‐stained with hematoxylin, dehydrated using a series of increasing concentrations of alcohol solutions and xylene, and sealed with cover slides. Images were captured under a light microscope at a magnification of ×40.

Expression in tissues was stratified as follows: (a) 0, <10%; (b) 1, 10–25%; (c) 2, 25–50%; (d) 3, 50–75%; and (e) 4, >75%. The intensity of staining was divided into four grades (intensity scores): (a) 0, no staining; (b) 1, light brown; (c) 2, brown; and (d) 3, dark brown. Fzd7 and Wnt7b staining positivity was evaluated using IHC scores, which were calculated as: Overall IHC score =percentage score × intensity score. Thus, based on the percentage and intensity scores, target protein staining was classified into four groups: (a) IHC score ≤3, negative; (b) IHC score >3 and ≤6, weak; (c) IHC >6 and ≤9, moderate; and (d) IHC >9, strong.

### 2.4 Cell culture and spheroid formation culture assay

Human PDAC cell lines, AsPC‐1, Capan‐2, Panc‐1, and SW1990, were obtained from American Type Culture Collection. All cell lines were cultured in DMEM (Gibco; Thermo Fisher Scientific, Inc.) with 10% FBS (MRC Biotechnology Co. Ltd.; cat. no. CCS30009.02) and 100 U/ml penicillin at 37°C in a humidified incubator with 95% air and 5% CO_2_.

When the adherent cells were in the logarithmic growth stage, they were digested and resuspended, and the cell concentration was adjusted to 1×10^4^ cells/ml and inoculated in the complete MammoCult™ Human Medium (Stemcell Technologies, Inc.) using 6‐well ultra‐low attachment Surface Polystyrene culture plates (Corning, Inc.). After 10–14 days, we observed and imaged the morphology and quantity of pancreatic cell spheres using an inverted light microscope.

### 2.5 Western blotting

Cells were lysed using cold RIPA lysis buffer (Beyotime), and lysates were centrifuged at 10000 × g for 30 min at 4℃, retaining the supernatants. The total protein concentration was quantified using a BCA Protein Assay Kit (Beyotime, Institute of Biotechnology). Total proteins were loaded on an 8% SDS‐gel, resolved using SDS‐PAGE, and then transferred to a PVDF membrane. Membranes were blocked in 5% non‐fat dried milk in TBST for 2 h and then incubated with the specific primary antibodies overnight, using GAPDH as the loading control. Membranes were subsequently incubated with horseradish peroxidase‐coupled secondary antibody for 2 h. The gray value of bands was normalized to the respective GAPDH bands. The primary antibodies used were as follows: Fzd7 (1:1000; Abcam, cat. no. ab64636), Wnt7b (1:1000; R&D Systems, cat. no. AF3460), ABCG2 (1:1000; Cell Signaling Technology, Inc.; cat. no. 42078), β‐catenin (1:1000; Cell Signaling Technology, Inc.; cat. no. 9562), active β‐catenin (1:1000; Cell Signaling Technology, Inc.; cat. no. 8814), and GAPDH (1:10,000; ProteinTech Group, Inc.; cat. no. HRP‐60004).

### 2.6 Co‐immunoprecipitation

Cell lysates of Capan‐2 and Panc‐1 were centrifuged at 12,000 rpm at 4°C. A total of 40 μl supernatant was taken as normal sample and 20 μl protein A/G agarose beads (Santa Cruz Biotechnology, Inc.; cat. no. sc‐2003) was added to the remaining supernatant and incubated for 30 min. After incubating and centrifuging, samples were divided into two tubes. A total of 2 μg Fzd7 (Santa Cruz Biotechnology, Inc.; cat. no. sc‐293261) and 2 μg mouse IgG were added. To each sample, 20 μl protein A/G agarose beads was added, and the mixture was incubated overnight. The non‐specifically bound proteins were removed by washing the agarose beads. Subsequently, the samples were analyzed using western blotting, as described above.

### 2.7 RNA interference and transfection

Small interfering RNAs (siRNAs) targeting Fzd7 or Wnt7b and control siRNAs were provided by Nanjing KeyGen Biotech Co., Ltd. Capan‐2 and Panc‐1 cells were seeded and cultured in 6‐well plates, and when cells were 60–80% confluent, the siRNAs were added to the medium using Lipofectamine™ 3000 reagent (Invitrogen; Thermo Fisher Scientific, Inc.) according to the manufacturer's protocol. After 48 h of transfection, the subsequent experiments were performed. The sequences of the siRNAs targeting Fzd7 were as follows: siFzd7‐1, 5′‐GTTCGTCTACCTCTTCATA‐3′; siFzd7‐2, 5′‐AGTACCTGATGACCATGAT‐3′; and siFzd7‐3, 5′‐AGCCGTACCACGGAGAGAA‐3′. The sequences of the siRNAs targeting Wnt7b were as follows: siWnt7b‐1, 5′‐CCCACCTTCCTGCGCATCAAA‐3′; siWnt7b‐2, 5′‐GCGCCTCATGAACCTGCATAA‐3′; and siWnt7b‐3, 5′‐CGTGCGTTACGGCATCGACTT −3′.

Capan‐2 and Panc‐1 cells transfected with short hairpin (sh)RNA lentiviruses (Nanjing KeyGen Biotech Co., Ltd.) targeted Fzd7 (5′‐GTTCGTCTACCTCTTCATA‐3′) and Wnt7b (5′‐CGTGCGTTACGGCATCGACTT‐3′), and transfected cells were selected for using 2 μg/ml puromycin (Invitrogen; Thermo Fisher Scientific, Inc.) for 48 h post‐transfection. Cells showing healthy growth with Fzd7/Wnt7b stable knockdown were maintained in DMEM supplemented with 10% FBS and 2 μg/ml puromycin.

### 2.8 Immunofluorescence assay

Cells were grown on cover slips for 24 h, fixed using 4% paraformaldehyde solution, and then permeabilized using PBS containing 1% Triton‐100 for 10 min. Cells were blocked with 2% BSA prepared in PBS for 10 min. Cells were then incubated with primary antibodies (Fzd7; 1:200, Abcam, cat. no. ab64636; or Wnt7b, 1:1000, Abcam, cat. no. ab94915) at 4℃ overnight. Subsequently, cells were incubated with Alexa Fluor 488 goat anti‐rabbit IgG(H + L)‐conjugated secondary antibodies (1:1000) for 1 h. Cell nuclei were stained with DAPI (Beyotime Institute of Biotechnology) for 10 min in the dark. Fluorescence was visualized using a confocal laser scanning microscope (Leica Laser Technik GmbH).

### 2.9 Flow cytometry assay

The pancreatic cancer cells in the logarithmic growth stage were collected and centrifuged. The concentration of cells was adjusted to 1×10^6^/ml.

For analysis of stemness markers, cells (1×10^6^) were labeled with PE‐conjugated CD24 (BD PharMingen™, cat. no. 555428) and APC‐conjugated anti‐CD44 (BD PharMingen™, cat. no. 559942), incubated at 4℃ in the dark for 30 min. Cells were then centrifuged and resuspended in PBS at a concentration of 1×10^8^/ml, and subsequently sorted on a flow cytometer (BD Accuri C6 Plus; BD Biosciences).

For apoptosis and necrosis analysis, cells were cultured for 12 h prior to being treated with gem for a further 24 h. For flow cytometry analysis, cells were detached and labeled using an Annexin V‐PE/7‐AAD Apoptosis Detection kit (Nanjing KeyGen Biotech Co., Ltd.; cat. no. KGA1017) according to the manufacturer's protocol. Apoptotic and necrotic cell subsets were quantified using flow cytometry. A total of 2×10^4^ cells were analyzed per sample. Annexin V‐PE^−^/7‐AAD^−^ cells were considered as viable, Annexin V‐PE^+^/7‐AAD^−^ cells were considered as early apoptotic, and Annexin V‐PE^+^/7‐AAD^+^ cells were considered as late apoptotic and necrotic cell populations.

### 2.10 MTT

Cells were plated in 96‐well plates and incubated for different periods of time, after which 20 μl MTT solution was added to each well (Nanjing KeyGen Biotech Co., Ltd.; cat. no. KGA312). Following incubation for 4 h, 150 μl DMSO was added to solubilize the crystals for 20 min at room temperature and the absorbance at 570 nm was measured using an ELISA plate reader (Model 680; Bio‐Rad, Laboratories, Inc.).

### 2.11 Establishment of the gem‐resistant cells

SW1990 cells were continuously treated with a low concentration of gem to obtain gem‐resistant cell line. First, the IC_50_ of gem was measured for the parental cells using an MTT assay, then 1/8 of the IC_50_ dose for parental cells was used as the induction concentration. After 24 h, the culture medium was changed and cultured until the cells were 70–80% confluent, after which, they were passaged. Cells were then cultured in medium containing a higher concentration of gem (1/4 IC50). This process was repeated, until the concentration of gem being used was >IC50 and the cells remained healthy, at which point, cells were frozen. After 3 days, we thawed and cultured the cells with medium containing the IC_50_ concentration of gem. Cells were observed under a light microscope, if cells were still growing well, then the cell line established was considered gem‐resistant.

### 2.12 Statistical analysis

Data are presented as the mean ±standard deviation of three independent experiments. The correlation between Fzd7/Wnt7b expression and clinicopathological characteristics was analyzed using a χ^2^ test. Spearman's correlation analysis was conducted for analysis of IHC scores using SPSS version 22.0 (IBM Corp.). Survival analysis was performed using GraphPad Prism version 7.0 (GraphPad Software, Inc.). Continuous data were compared using a one‐way ANOVA followed by a Tukey's post hoc test for comparisons between multiple groups, or a paired t‐tests for comparison between two groups. *p* < 0.05 was considered to indicate a statistically significant difference.

## 3 RESULTS

### 3.1 Bioinformatics analysis of Fzd7 and Wnt7b expression in pancreatic cancer

**FIGURE 1:**
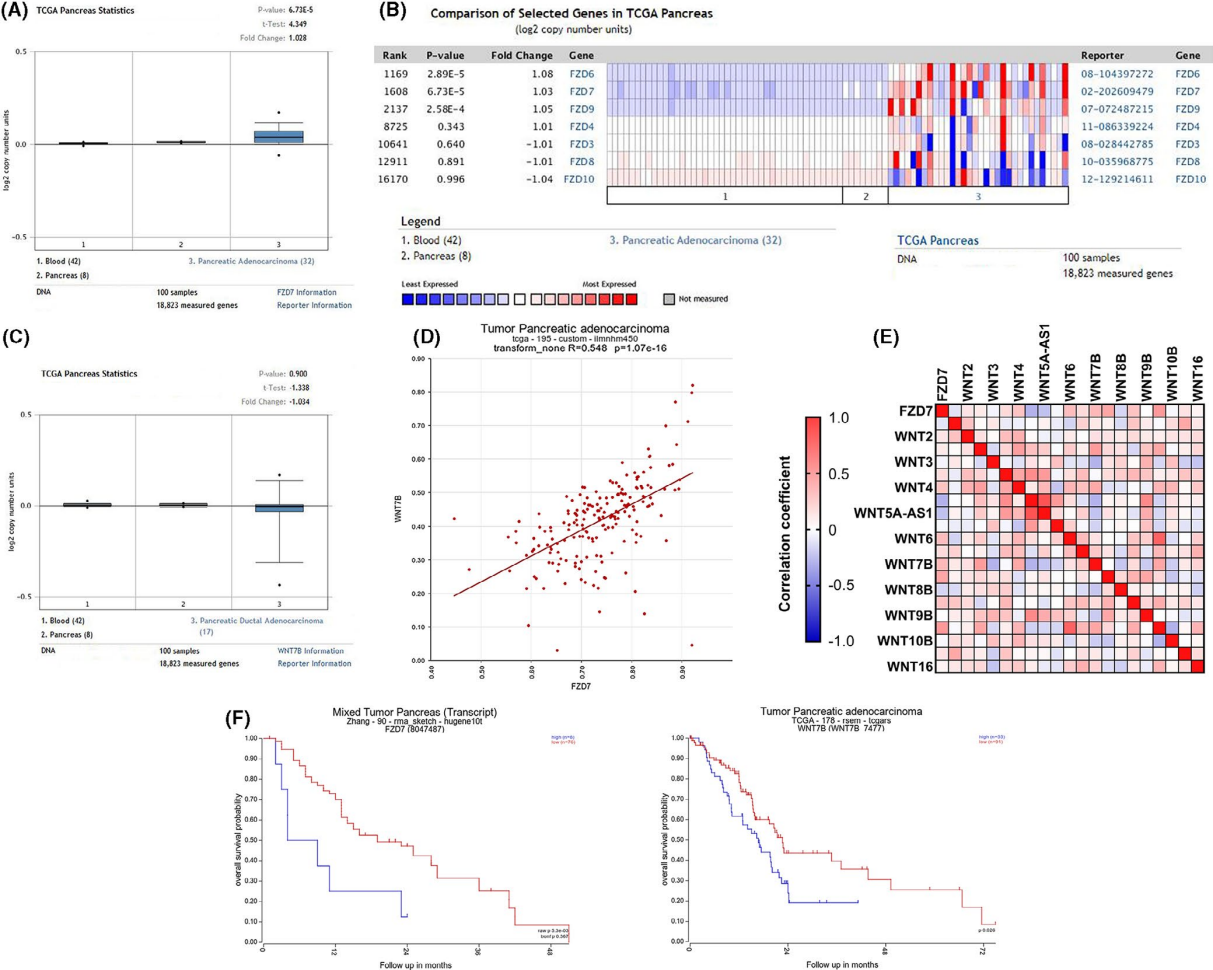
Data from the ONCOMINE database showed that the gene expression of Fzd7 was significantly higher in pancreatic adenocarcinoma compared with the normal pancreatic tissues (TCGA Pancreas Statistics, *p* = 6.73e^−5^) (A), and Fzd7 was significantly overexpressed in pancreatic adenocarcinoma compared with other Frizzled receptors (B). Gene expression of Wnt7b was not significantly different between pancreatic carcinoma and the normal pancreatic tissues as shown in the TCGA Pancreas Statistics datasets (C). Co‐expression analysis of Fzd7 and Wnt7b was performed in R2 database using the tcga‐195 dataset, which showed that Wnt7b was significantly associated with Fzd7 (*r* = 0.548, *p* = 1.07e^−16^) (D). Analysis on the data from CCLE indicates that Wnt7b expression was associated with Fzd7 at a relatively high level compared with other Wnt proteins (E). Kaplan–Meier analysis in the R2 database indicated that pancreatic cancer patients with higher levels of Fzd7 had a worse overall survival probability in the Zhang‐90 dataset (*p* = 5.3e^−3^), and high levels of Wnt7b also conferred a poorer prognosis in the TCGA‐178 dataset (*p* = 6.2e^−3^) (F)

First we investigated the expression of Fzd7 and Wnt7b by querying the ONCOMINE database. Most datasets suggested that Fzd7 expression was significantly higher in pancreatic cancer compared with normal pancreatic tissue. For example, in one microarray expression studies, the expression of Fzd7 was significantly higher in the PDAC tissues compared with the normal pancreatic tissues (Figure 1A). And in the ONCOMINE database, Fzd7 was significantly overexpressed in pancreatic adenocarcinoma compared with other Frizzled receptors (Figure 1B). Expression of Wnt7b did not differ significant difference between the pancreatic carcinoma and the normal pancreatic tissues (Figure 1C). Whereas co‐expression analysis of Fzd7 and Wnt7b was performed in the R2 database using “TCGA‐195” dataset, which showed that Wnt7b was significantly associated with Fzd7 (*r* = 0.548, *p* = 1.07e^−16^), the difference was statistically significant (Figure 1D). Based on the data from CCLE, we analyzed the correlation between the expression of Fzd7 and different Wnt proteins in various pancreatic cancer cell lines, and found that Wnt7b was more significantly associated with Fzd7 compared with other Wnt proteins relatively (Figure 1E).

Analysis of the clinical prognosis of pancreatic cancer patients using Kaplan–Meier analysis in the R2 database revealed that pancreatic cancer patients with high levels of Fzd7 had a worse overall survival probability, and high levels of Wnt7b were also associated with a poor prognosis for pancreatic cancer patients (Figure 1F).

### 3.2 Expression of Fzd7 and Wnt7b in surgical specimens and cell lines

A total of 37 surgical samples of PDAC were included in the present study. As receptors and ligands, Fzd7 and Wnt7b were co‐expressed in the immunohistochemical staining of pancreatic cancer tissues to different degrees (Figure 1A). The Fzd7 expression in cancer tissues was significantly higher compared with the paracancerous tissues. The immunohistochemical assay showed that 28 cases (75.7%) were positive for Fzd7 among the 37 cancer tissue samples and seven cases (18.9%) were positive for Fzd7 in the 37 paracancerous tissues, and the IHC scores were 6.2 ± 3.4 and 2.9 ± 3.1, respectively (*p* < 0.05). Additionally, 18 cases (48.6%) were positive for Wnt7b among the 37 cancer tissue samples and 15 cases (40.5%) were positive for Wnt7b among the 37 paracancerous tissue samples, and the IHC scores were 3.5 ± 1.7 and 3.2 ± 2.7, respectively, with no statistically significant difference.

**FIGURE 2:**
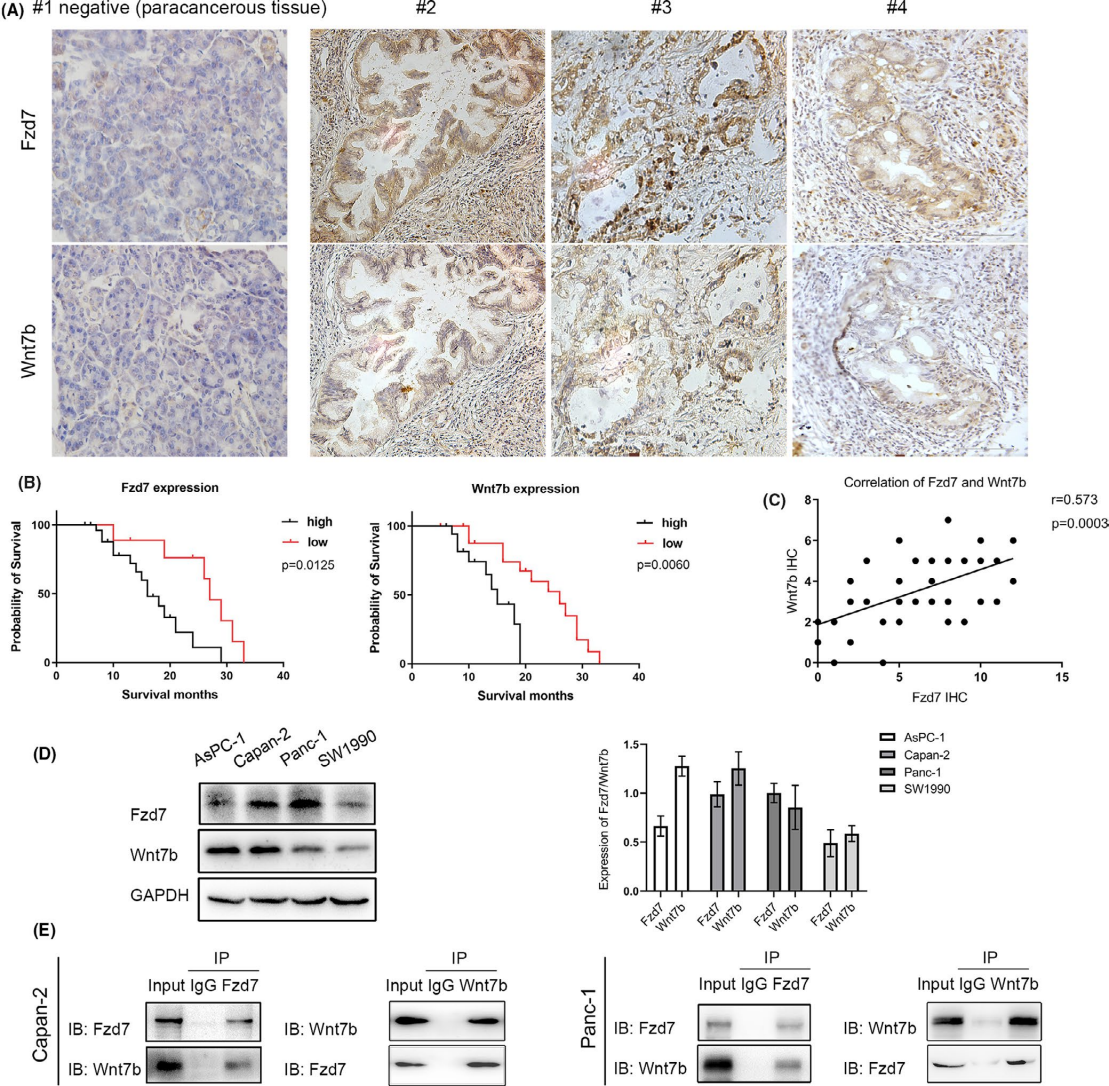
Fzd7 and Wnt7b exhibited colocalization in the immunohistochemical staining of pancreatic cancer tissues to different degrees (A). The Fzd7 expression in cancer tissues was significantly higher compared with the paracancerous tissues. Survival analysis showed that the survival probability of Fzd7 and Wnt7b in the high expression group was lower than that in the low level group (*p* < 0.05, *p* < 0.01) (B). Spearman's correlation analysis of the Fzd7 and Wnt7b levels in all 37 tissue samples (correlation coefficient was 0.573, *p* = 0.0003) (C). A total of four PDAC cells, AsPC‐1, Capan‐2, Panc‐1, and SW1990, exhibited different expression levels of Fzd7/Wnt7b (D). In the Co‐IP, Fzd7 and WB were precipitated together in Capan‐2 and Panc‐1 (E). PDAC, pancreatic ductal adenocarcinoma

We analyzed the survival probability of 37 cases based on Fzd7 and Wnt7b expression, and the results showed that the survival probability of Fzd7 and Wnt7b in the high expression group was lower compared with the low expression group, and the difference was statistically significant (*p* < 0.05 and *p* < 0.01, respectively; Figure 2B). In addition, we performed correlation analysis between the Fzd7 and Wnt7b levels of all the tissue samples; the Spearman's correlation coefficient was 0.573, *p* = 0.0003 (Figure 2C).

We selected four PDAC cell lines, AsPC‐1, Capan‐2, Panc‐1, and SW1990, for western blotting, and the results indicated that the four cell lines all had different levels of Fzd7/Wnt7b expression (Figure 2D). In order to further clarify whether Fzd7 and Wnt7b exhibited properties in cells dependent on each other, Capan‐2 and Panc‐1 cells, which have relatively high levels of Fzd7 expression normally, were selected for Co‐IP (Figure 2E). We precipitated using Fzd7 antibody, and then processed using western blotting using the Wnt7b antibody. The results showed that the Wnt7b bands were clearly visible in the input. Then we precipitated using Wnt7b antibody, and processed using western blotting using the Fzd7 antibody, and obtained the same results. This demonstrated that Fzd7 bound to Wnt7b in living cells. Therefore, we hypothesized that Wnt7b was an important ligand which exhibited synergistic action with Fzd7 in living pancreatic cancer cells.

### 3.3 Fzd7/Wnt7b knockdown can decrease the CSC phenotype of PDAC cells

**FIGURE 3:**
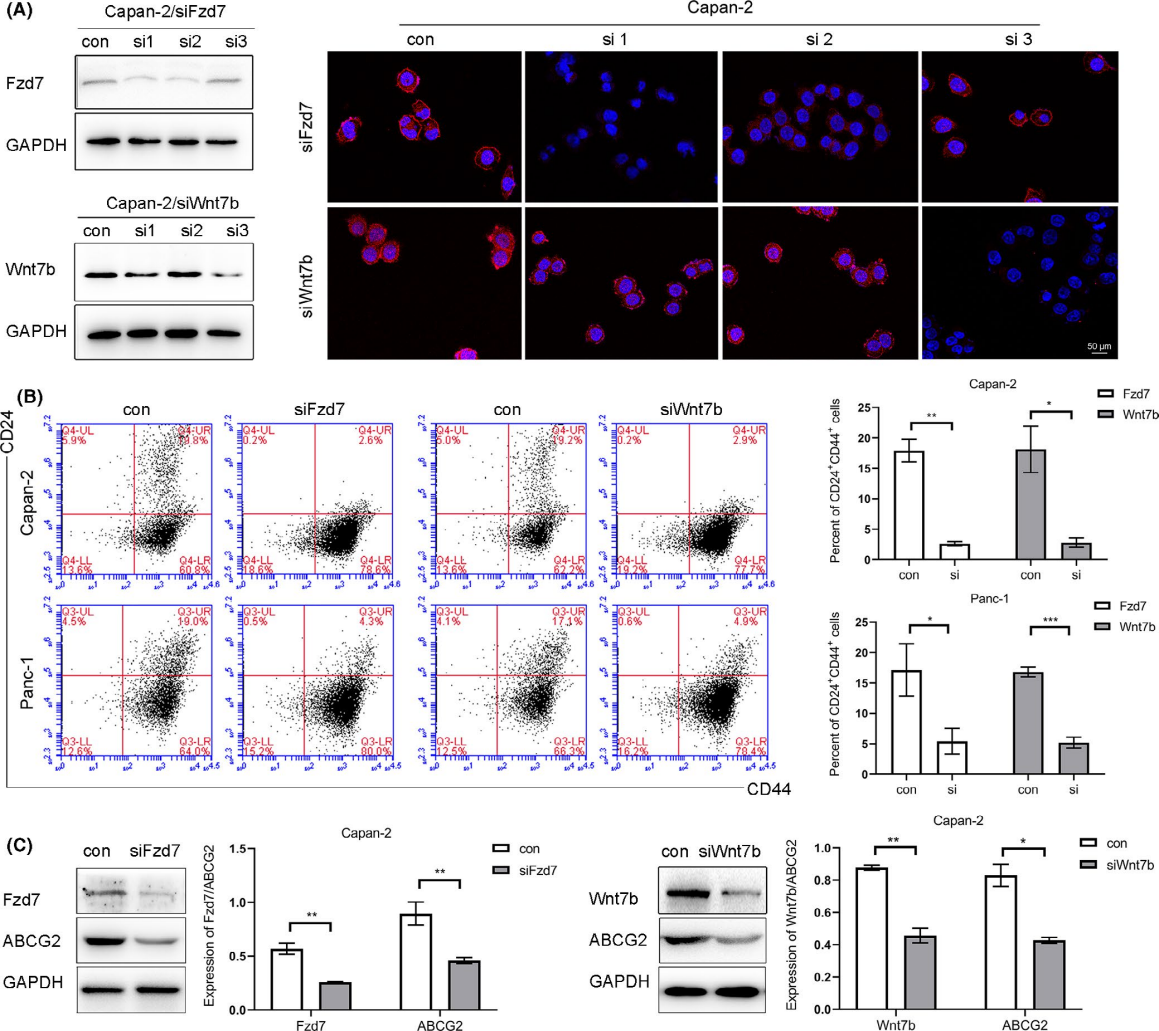
Western blotting and immunofluorescence analysis were used to assess the effects of transfection of siRNA (A). Flow cytometry showed that CD24^+^CD44^+^ cells accounted for about 17.9 ± 1.9% and 17.1 ± 4.3% in Capan‐2 and Panc‐1 cell lines, respectively. This was reduced to 2.6 ± 0.4% and 5.4 ± 2.1% after transfection with the siRNA‐Fzd7; and 18.1 ± 2.8%/16.8 ± 0.8 and 2.8 ± 0.75%/5.2 ± 0.9% when transfected with the siRNA‐Wnt7b in the Capan‐2 and Panc‐1 cells, respectively; the differences were statistically significant (B). Western blotting showed that the ABCG2 levels were reduced when Fzd7/Wnt7b expression was knocked down in the Capan‐2 and Panc‐1 cells; the difference was statistically significant (C). *p < 0.05, **p < 0.01, ***p < 0.001. siRNA, small interfering RNA

Combination of several markers may enhance the purity of isolated CSCs. For example, Li C et al. reported that CD44^+^CD24^+^ESA^+^ cells account for ~0.2–0.8% of all pancreatic cancer cells possessing properties of stem cells, which exhibit a 100‐fold increased tumorigenic potential compared with CD44^−^CD24^−^EpCAM^−^ cells.^8^ In a study by Jianhui Zhu et al, stem‐like cells were enriched in the CD24^+^CD44^+^ cell subset, 12.15% of Panc‐1 cells were double positive for both CD24 and CD44, and CD24^+^ cells were all CD44^+^ cells.^29^ In the present study, we used flow cytometry to detect the CD24^+^CD44^+^ cell subset. The results showed that CD24^+^CD44^+^ cells accounted for 17.9 ± 1.9% and 17.1 ± 4.3% in Capan‐2 and Panc‐1, respectively, whereas the percentage of CD24^+^CD44^+^ cells was reduced to 2.6 ± 0.4% and 5.4 ± 2.1% after transfection of siRNAs targeting Fzd7; the results were 18.1 ± 2.8%/16.8 ± 0.8 and 2.8 ± 0.75%/5.2 ± 0.9% when treated with siRNAs targeting Wnt7b in Capan‐2 and Panc‐1 (Figure 3B). In addition, western blotting analysis showed that the ABCG2 expression was reduced with the decrease of Fzd7/Wnt7b levels in Capan‐2 and Panc‐1 (Figure 3C), which exhibited relatively high levels of Fzd7/Wnt7b expression normally (Figure 2C).

### 3.4 Effects of Fzd7/Wnt7b on sphere formation of PDAC cells

**FIGURE 4:**
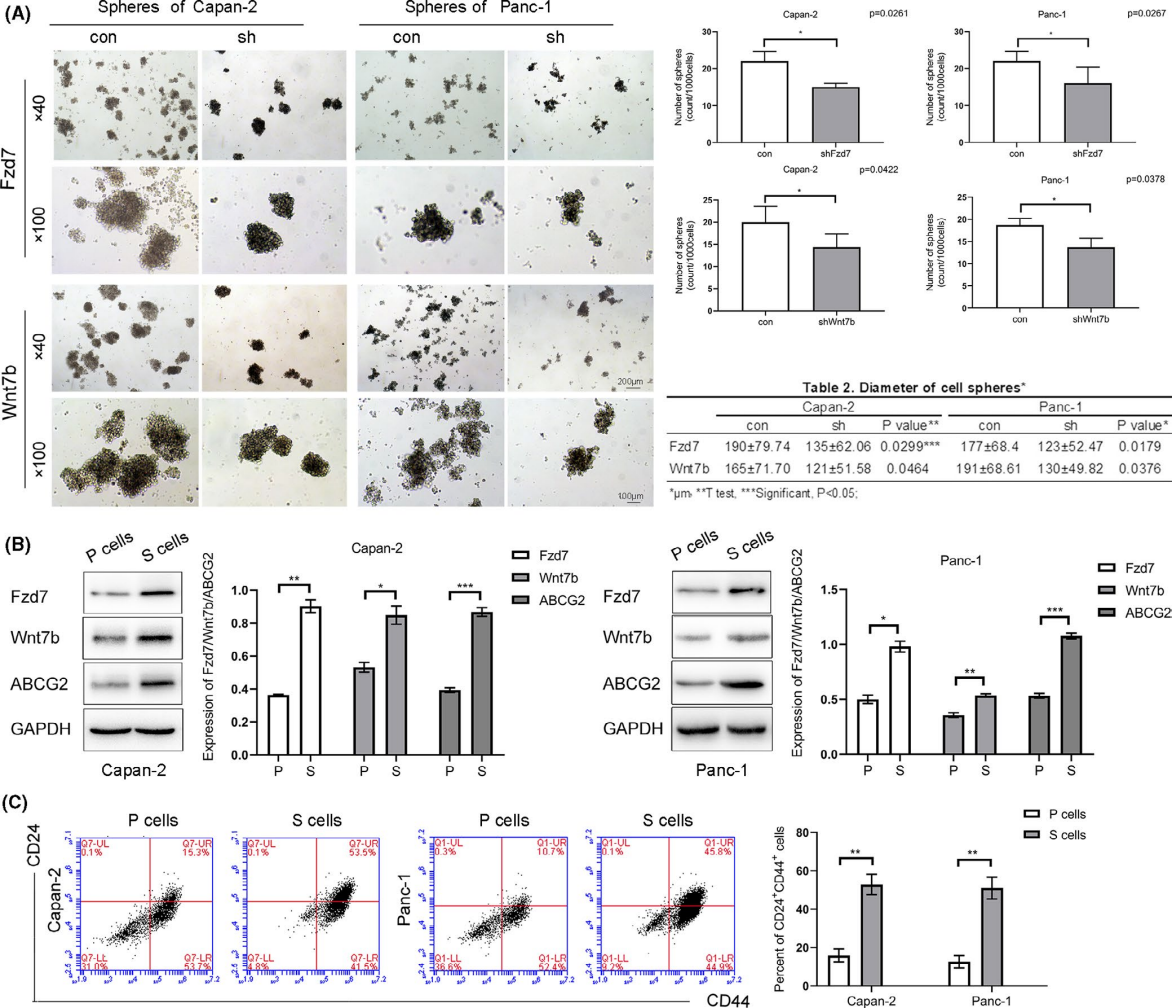
After silencing Fzd7/Wnt7b expression using lentiviral shRNA targeting Fzd7/Wnt7b, spheroid formation per 1000 cells decreased from 22.0 ± 2.6/20.0 ± 3.6 (con) to 15.0 ± 1.0/14.3 ± 3.1 in the Capan‐2 cells, and from 22.0 ± 2.6/18.7 ± 1.5 (con) to 16.0 ± 4.4/13.7 ± 2.1 in the Panc‐1 cells (shFzd7/Wnt7b), the difference was statistically significant. The diameter of the cell spheres decreased from 190.74 ± 79.74/165 ± 71.7 μm (con) to 135 ± 62.06/121 ± 51.58 μm in Capan‐2 cells, and from 177 ± 68.4/191 ± 68.61 μm (con) to 123 ± 52.47/130 ± 49.82 μm in the Panc‐1 cells (shFzd7/Wnt7b), the difference was statistically significant (A). In Capan‐2 and Panc‐1, S cells expressed higher levels of Fzd7, Wnt7b, and ABCG2, the difference was statistically significant (B), and the proportion of CD24^+^CD44^+^ cells in the Capan‐2 and Panc‐1 cells increased from 15.9 ± 3.4% and 12.6 ± 3.2% in P cells to 52.9 ± 5.3% and 51.0 ± 5.7% in S cells, respectively, the difference was statistically significant (C). *p < 0.05, **p < 0.01, ***p < 0.001. shRNA, short hairpin

CSCs are immortal cells accounting for a very small proportion of the tumor tissue, and they possess strong proliferative capacity, and can form non‐adherent cell spheroids in vitro; the PDAC cell lines can also form spheres.^30^, ^31^, ^32^ In Capan‐2 and Panc‐1 cells, after silencing Fzd7/Wnt7b expression using lentiviral shRNAs, the number and diameter of cell spheroids formed were significantly reduced (Figure 4A). In addition, western blotting was performed on the lysates obtained from spheroids (S cells) and adherent cultured parental cells (P cells). The former exhibited higher levels of Fzd7, Wnt7b, and ABCG2 (Figure 4B). Additionally, the proportion of CD24^+^CD44^+^ cells in the S cells was notably higher than the P cells, which was increased from 15.9 ± 3.4% and 12.6 ± 3.2% to 52.9 ± 5.3% and 51.0 ± 5.7% in Capan‐2 and Panc‐1 cells, respectively (Figure 4C).

### 3.5 Fzd7/Wnt7b can affect gem resistance of PDAC cell lines

**FIGURE 5:**
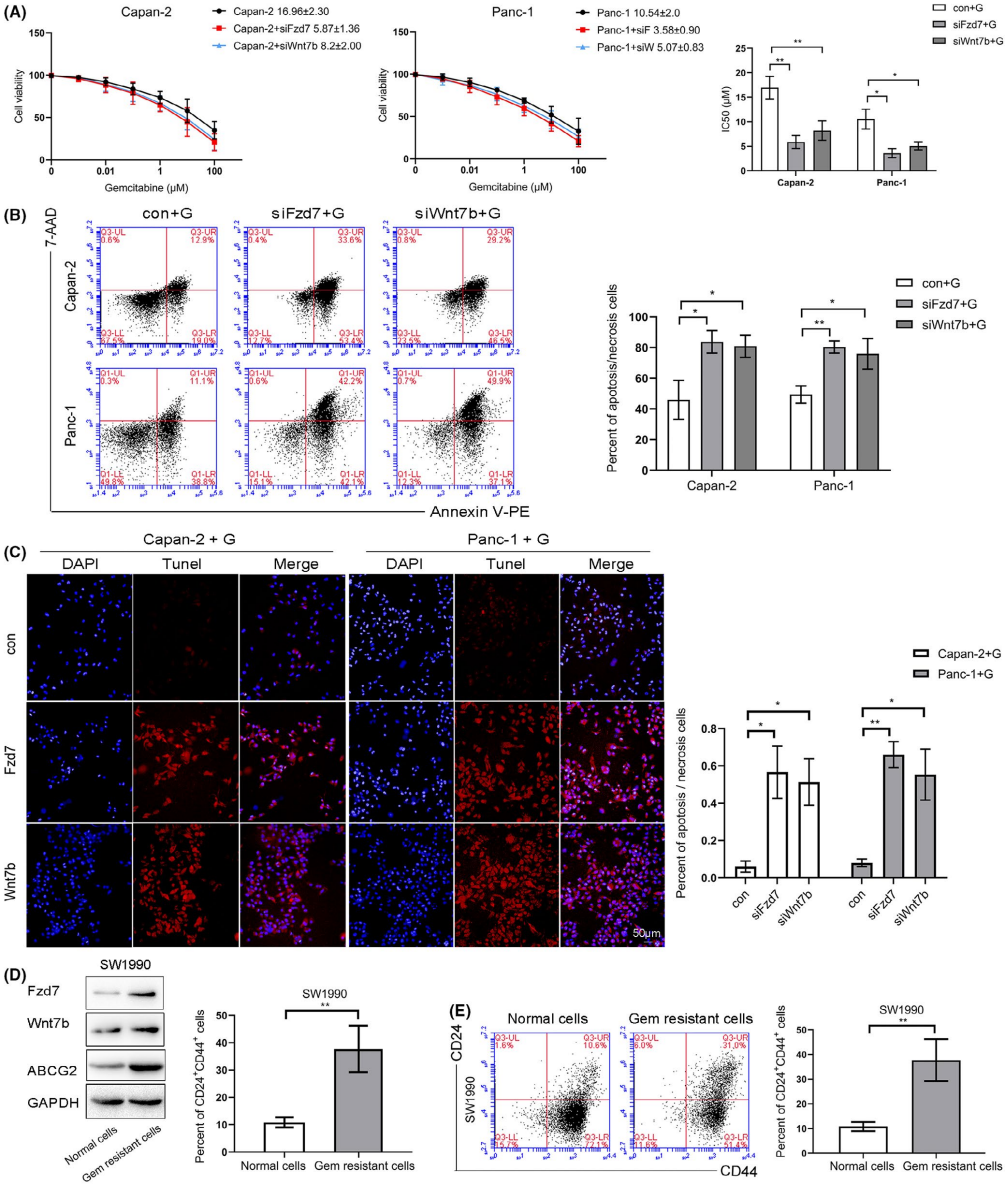
The IC_50_ value of gem in the Capan‐2 cells was 16.96 ± 2.30 μM, which decreased to 5.87 ± 1.36 μM and 8.20 ± 2.0 μM after transfection with siRNA targeting Fzd7 and Wnt7b, receptively, the difference was statistically significant. In the Panc‐1 cells, the IC_50_ value of gem decreased from 10.54 ± 2.0 μM to 3.58 ± 0.90 μM and 5.07 ± 0.83 μM after transfection with siRNA targeting Fzd7 and Wnt7b, receptively, the difference was statistically significant (A). Compared with the control group, after knockdown of Fzd7/Wnt7b expression, cells were treated with gem of the IC_50_ dose, the proportion of late apoptotic and necrotic cells was significantly increased, from 13.4 ± 1.7% (con) to 29.6 ± 1.7% (siFzd7) and 35.3 ± 2.7% (siWnt7b) in the Capan‐2 cells, and from 9.0 ± 3.4% (con) to 28.1 ± 2.2% (siFzd7) and 28.9 ± 3.9% (siWnt7b) in the Panc‐1 cells (all *p* < 0.05) (B). The quantity of TUNEL‐positive cells increased significantly from 6.0 ± 3.0% (con) to 56.7 ± 14.0% (siFzd7) and 51.3 ± 12.5% (siWnt7b) in the Capan‐2 cells, and from 8.0 ± 2.0% (con) to 66.0 ± 7.0% (siFzd7) and 55.3 ± 13.7% (siWnt7b) in the Panc‐1 cells, respectively (*p* < 0.05) (C). The Fzd7/Wnt7b and ABCG2 levels of gem‐resistant cells were significantly higher compared with the parental cells, and the difference was statistically significant (D). The proportion of CD24^+^CD44^+^ cells was significantly increased in the gem‐resistant cells, from 15.9 ± 3.4% in parental cells to 52.9 ± 5.3% in gem‐resistant cells, and the difference was statistically significant (E). *p < 0.05, **p < 0.01, ***p < 0.001. siRNA, small interfering RNA

The IC_50_ value of gem in Capan‐2 was 16.96 ± 2.30 μM, while it was 5.87 ± 1.36 μM or 8.20 ± 2.0 μM when expression of Fzd7 or Wnt7b was knockdown using siRNA, the difference was statistically significant. In Panc‐1, the IC_50_ value of gem is 10.54 ± 2.0 μM compared with 3.58 ± 0.90 μM and 5.07 ± 0.83 μM, and the difference was statistically significant also (Figure 5A). We treated Fzd7/Wnt7b knockdown Capan‐2/Panc‐1 using a gem dose of the IC_50_ concentration, to detect the percentage of necrotic and late apoptotic cells in the flow cytometry and TUNEL assays, and observe whether there were differences following Fzd7/Wnt7b knockdown. Compared with the control group, after Fzd7/Wnt7b levels were knocked down, the proportion of late apoptotic and necrotic cells detected by flow cytometry was significantly increased (*p* < 0.05; Figure 5B), and the quantity of TUNEL‐positive cells was also significantly increased (*p* < 0.05; Figure 5C). These results suggest that gem resistance was reduced following Fzd7/Wnt7b depletion.

As the previous results showed that the proportion of CD24^+^CD44^+^ cells and ABCG2 protein expression levels were both decreased following knockdown of Fzd7/Wnt7b in Capan‐2 and Panc‐1 cells, we speculated that the reduction of gem resistance by Fzd7/Wnt7b was achieved by decreasing the proportion of PCSCs. To confirm this hypothesis, gem‐resistant cells were established using the SW1990, which express relatively low levels of Fzd7/Wnt7b normally (Figure 2D). Western blotting showed that Fzd7/Wnt7b and ABCG2 levels of gem‐resistant cells were significantly higher compared with the parental normal cells (Figure 5D), and the proportion of CD24^+^CD44^+^ cells was significantly increased in gem‐resistant cells compared with the parental normal cells (Figure 5E). It was speculated that Fzd7/Wnt7b can increase the proportion of the CSC subset, increasing the drug resistance of the cell lines.

### 3.6 Fzd7/Wnt7b function via the classical Wnt pathway

**FIGURE 6:**
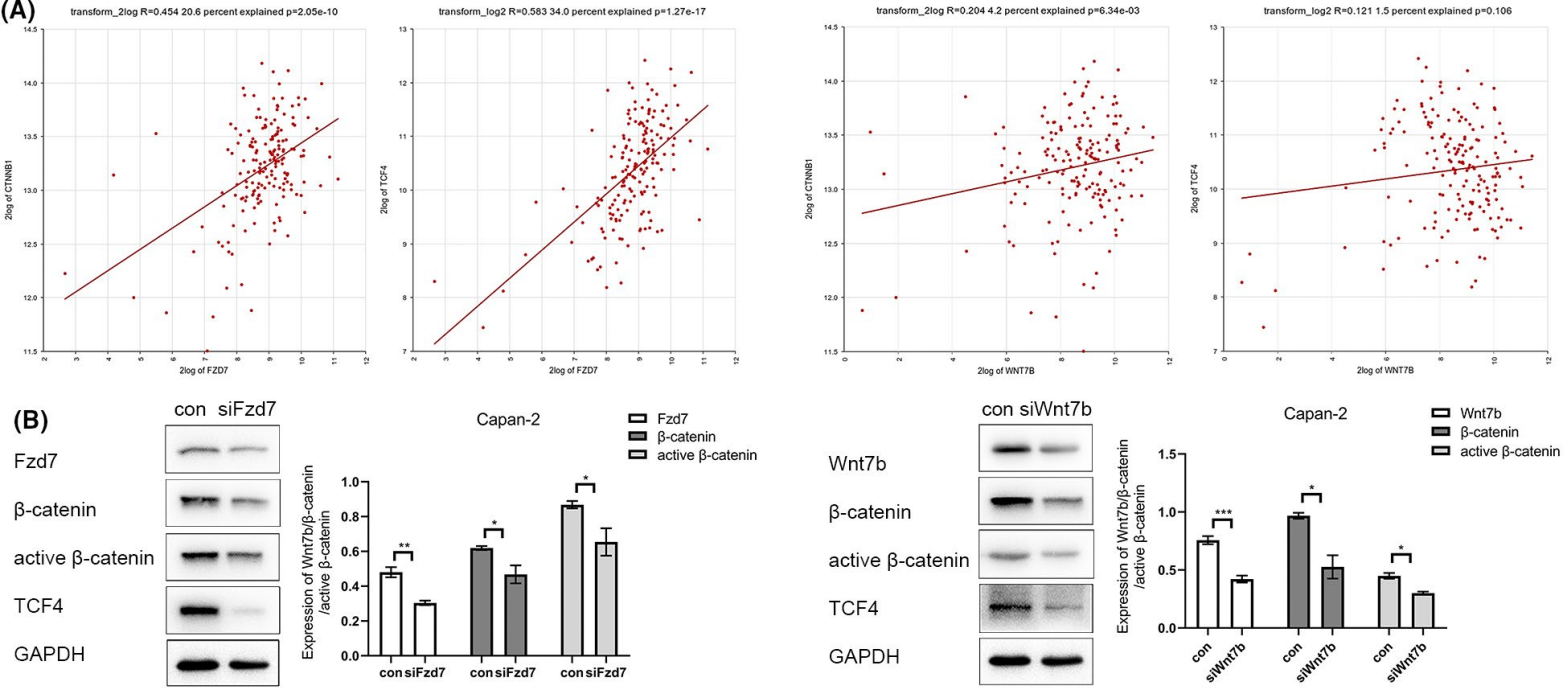
CTNNB1 expression is positively correlated with Fzd7 and Wnt7b gene expression in the TCGA‐178 dataset of the R2 database (*r* = 0.454, *p* = 2.05e^−10^ and *r* = 0.204, *p* = 6.34e^−03^, respectively); TCF4 expression is positively correlated with Fzd7 and Wnt7b gene expression in the TCGA‐178 dataset of the R2 database (*r* = 0.583, P = 1.27e^−17^ and *r* = 0.121, *p* = 0.106, respectively) (A). In the Capan‐2 cells, total β‐catenin, active β‐catenin, and TCF4 levels decreased following Fzd7/Wnt7b knockdown, and the difference was statistically significant (B). *p < 0.05, **p < 0.01, ***p < 0.001

β‐catenin is encoded by the CTNNB1 gene. TCF4 acts downstream in Wnt signaling, which binds to Wnt response elements to provide docking sites for β‐catenin to promote the transcription of target genes upon activation of Wnt signaling. Based on the R2 database, CTNNB1 expression was positively correlated with Fzd7/Wnt7b gene expression (*r* = 0.454, *p* = 2.05e^−10^/*r* = 0.204, *p* = 6.34e^−03^), and TCF4 expression was positively correlated with Fzd7/Wnt7b gene expression also (*r* = 0.583, *p* = 1.27e^−17^/*r* = 0.121, *p* = 0.106) (Figure 6A). In the Capan‐2 cells, total β‐catenin, active β‐catenin, and TCF4 levels decreased following Fzd7/Wnt7b knockdown using siRNA. This suggested that β‐catenin/TCF4 acted downstream of Fzd7/Wnt7b, and that Fzd7/Wnt7b functions via the classical Wnt pathway (Figure 6B).

## 4 DISCUSSION

Using specific cell surface markers is a typical approach to identify and isolate CSCs in different types of tumors in vitro.^33^ It has been reported that Fzd7 can positively regulate the expression of CD24, CD44, ABCG2, and other CSC phenotypic molecules in AGS and HGC27 cells.^34^ However, in the Wnt signaling pathway, the Wnt ligand binds to the Frizzled receptor, and this initiates the downstream molecular cascade, and binding of Fzd7 with Wnt ligand is required for induction of the synergistic effects. Wnt7b is the most effective activator of the Wnt/β‐catenin pathway in pancreatic adenocarcinoma.^35^ Correlation analysis between Fzd7 and Wnt7b in the R2 database and CCLE was performed, and the results showed that there was significant positive correlation between the expression of the two proteins in pancreatic tumors (Figure 1D and E). To verify whether Fzd7 bound to Wnt7b in living cells, we conducted Co‐IP on Capan‐2 and Panc‐1 cells, which exhibit relatively high protein expression levels of Fzd7/Wnt7b, and the results were positive (Figure 2D). The IHC analysis also supported this result, as both Fzd7 and Wnt7b exhibited colocalization in the staining analysis (Figure 2A). Protein expression of Fzd7/Wnt7b in pancreatic cancer tissues is a prerequisite for further biological functional studies.

By mining the Oncomine database, we found that the expression levels of Fzd7 in pancreatic malignant tumors were higher than that in the normal pancreatic tissues (Figure 1A and B). Survival analysis using the R2 database showed a negative correlation between the expression levels and survival probability, implying that these two proteins are carcinogenic molecules (Figure 1F). In order to determine whether the protein levels of Fzd7/Wnt7b in PDAC altered the expression of CSC phenotypic molecules, siRNAs were used to knockdown Fzd7/Wnt7b expression in the Capan‐2 and Panc‐1 cells, which had relatively higher levels of Fzd7/Wnt7b. The results showed that when the protein expression levels of Fzd7/Wnt7b decreased, the proportion of CD24^+^CD44^+^ cells (Figure 3B) and the protein levels of ABCG2 decreased simultaneously (Figure 3C). These results suggest that the proportion of CSCs may be regulated by Fzd7/Wnt7b.

In addition to the detection of CSC phenotypic molecules, enrichment of CSCs by cell suspension culture and detection of side population (SP) cells are often reported to be used to identify and isolate CSC subsets in vitro.^36^ Cell suspension cultures were initially used to culture stem cells, such that stem cells could grow well in suspension culture conditions and grow into cell spheres, whereas normal differentiated cells can only grow by adhering to the bottom. We treated Capan‐2 and Panc‐1 cells with lentiviruses to silence Fzd7/Wnt7b expression, and then performed suspension culture for 10–14 days to observe whether the sphere‐forming ability was affected. The results indicated that cell sphere‐forming ability was significantly inhibited by Wnt7b/Fzd7 silencing. This result shows that Fzd7/Wnt7b can regulate the proliferation and amplification ability of CSCs. On the other hand, S cells had higher levels of Fzd7, Wnt7b, and ABCG2 expression compared with adherent P cells, and CD24^+^CD44^+^ cells in the S cells accounted for a higher percentage than the adherent P cells (Figure 4). That is to say, when CSCs were enriched in S cells, the levels of Fzd7/Wnt7b in S cells were increased as the percentage of CD24^+^CD44^+^ cells increased as well as the ABCG2 levels. This suggests that the protein expression levels of Fzd7/Wnt7b in CSCs were higher compared with the normal cells. Therefore, the levels of Fzd7/Wnt7b may be an important factor that can regulate the proportion of CSC subsets in pancreatic cancer cells.

The side population cells (SP cells) that can exclude the DNA dye Hoechst 33342 extracellularly are considered to possess stem‐like features in tumors.^37^, ^38^, ^39^ Zhou J et al. treated Panc‐1 with Hoechst 33342 showing that SP cells accounted for 2.1–8.7% of cells (median 3.3%).^40^ ABCG2 serves as the determinant of the specific phenotype of SP cells and is responsible for excluding the DNA dye Hoechst 33342.^17^, ^37^, ^38^, ^39^

The mRNA expression of the Bcrp1 gene (which encodes the ABCG2 protein) is high in primitive murine hematopoietic stem cells, and is notably downregulated as the cells differentiate.^17^ Reculturing of SP cells in vitro generated both SP cells and non‐SP cells, whereas reculturing non‐SP cells generated predominantly non‐SP cells.^41^ These data suggest that ABCG2^+^ cells or SP cells were undifferentiated or relatively lowly differentiated cells, possessing potent differentiation capacity, consistent with the characteristics of the capacity of CSCs to differentiate into multiple different types of cells. Thus, the SP cell subset may have a high degree of coincidence with CSCs; that is, SP cells may contain CSC subsets or vice versa.^42^, ^43^

Since ABCG2 is responsible for drug resistance of cancer cells,^17^, ^18^, ^19^ and CSCs express high levels of ABC transporters, particularly ABCG2,^20^ so we speculate that Fzd7/Wnt7b knockdown reduced the gem resistance by reducing the proportion of CSCs. In gem‐resistant SW1990 cells, the expression of Fzd7/Wnt7b and ABCG2 and the proportion of CD24^+^CD44^+^ were increased simultaneously, suggesting that the primary population of gem‐resistant SW1990 cells was composed of CSCs and Fzd7/Wnt7b contributed to the acquisition of gem resistance, similar to the role of Fzd7/Wnt7b in the S cells. In the process of establishing drug‐resistant cells, ordinary cancer cells that did not express or expressed low levels of ABCG2 were killed by gem, whereas the CSC subset expressing high levels of ABCG2 were selected, forming the drug‐resistant cell line. Therefore, the CSC subset is an important mediator of drug resistance in cell lines and tumors.

In addition, Ishiwata T et al. showed that ABCG2^+^ cells account for the majority of S cells, and these ABCG2^+^ cells possess stem‐like properties and resistance in pancreatic cancer cells.^32^ Tao Yin et al. also confirmed that pancreatic cancer S cells exhibited increased expression of ABCG2 and resistance to gem.^31^ This phenomenon was also observed in liver cancer and gallbladder cancer cells in vitro.^44^, ^45^ Since ABCG2 is highly expressed in S cells, SP cells, and CSCs, we also speculate that S cells, SP cells, or CSCs will exhibit a high degree of overlap with drug‐resistant cells.

In our study, total β‐catenin, active β‐catenin, and TCF4 expression decreased following knockdown of Fzd7/Wnt7b, and combined with the Co‐IP and IHC results, it is reasonable to consider that Wnt7b is an important ligand for Fzd7. The two serve a synergistic role in PDAC cells and induce stemness or increase the proportion of CSCs via the classical Wnt signaling pathway.

## 5 CONCLUSION

Fzd7/Wnt7b is an important regulator of the stemness and drug resistance of pancreatic cancer cells, which may be due to PCSC subset regulation.

## ETHICS APPROVAL STATEMENT

This study was approved by The Ethical Committee of The First Hospital of China Medical University [approval no. (2015)100].

## CONFLICT OF INTEREST

The authors declare that they have no competing interests.

## Data Availability

The data that support the findings of this study are available from the corresponding author upon reasonable request.
